# CD47-Targeted Therapy in Cancer Immunotherapy: At a Crossroads of Promise and Challenge

**DOI:** 10.32604/or.2025.071708

**Published:** 2025-10-22

**Authors:** Xuejun Guo, Yilin Fu, Natalia Baran, Wenxue Ma

**Affiliations:** 1 Department of Hematology, Puyang Oilfield General Hospital Affiliated to Henan Medical University, Puyang, 457001, China; 2 Puyang Cell Therapy Engineering Technology Research Center, Puyang, 457001, China; 3 China Medical University-the Queen’s University of Belfast Joint College, Shenyang, 110112, China; 4 Department of Hematology and Central Hematology Laboratory, Inselspital, Bern University Hospital, University of Bern, Bern, 3012, Switzerland; 5 Department of Internal Medicine, Sanford School of Medicine, University of South Dakota, Sioux Falls, South Dakota, Sioux Falls, SD 57104, USA; 6 Department of Medicine, Sanford Stem Cell Institute, Moores Cancer Center, University of California San Diego, La Jolla, San Diego, CA 92093, USA

**Keywords:** Cluster of differentiation 47, cancer immunotherapy, macrophages, immune evasion, combination therapy

## Abstract

Cluster of differentiation 47 (CD47), an immune checkpoint commonly referred to as the “don’t eat me” signal, plays a pivotal role in tumor immune evasion by inhibiting phagocytosis through interaction with signal regulatory protein alpha (SIRPα) on macrophages and dendritic cells (DCs). Although early enthusiasm drove broad clinical development, recent discontinuations of major CD47-targeted programs have prompted re-evaluation of its therapeutic potential. The purpose of this commentary is to contextualize the setbacks observed with first-generation CD47 inhibitors and to highlight strategies aimed at overcoming their limitations. Clinical challenges, including anemia, thrombocytopenia, suboptimal pharmacokinetics, and limited single-agent efficacy, underscore the need to develop safer, more selective approaches. Emerging next-generation strategies, such as SIRPα-directed agents, bispecific antibodies, and conditionally active therapeutics, are designed to enhance safety and tumor selectivity and reduce systemic toxicity. In addition, spatial profiling and biomarker-driven patient selection are advancing toward guiding rational therapeutic combinations, including with “eat-me” signals (e.g., calreticulin [CALR]) or DNA damage response therapies (e.g., poly(ADP-ribose) polymerase [PARP] inhibitors). Rather than signaling failure, these developments underscore the need for precision, context-specific applications, and adaptive trial designs to realize the durable therapeutic promise of CD47 blockade in cancer immunotherapy.

## 1 Introduction

The immune checkpoint molecule cluster of differentiation 47 (CD47), often described as the “don’t eat me” signal, has gained increasing attention for its role in promoting tumor immune evasion [1,2]. CD47 is broadly expressed on normal cells but is frequently upregulated in cancer, including hematologic malignancies and solid tumors, where it engages signal regulatory protein alpha (SIRPα) on macrophages and dendritic cells (DCs) [3,4]. This interaction transmits a potent anti-phagocytic signal that inhibits the clearance of tumor cells, allowing them to persist despite immunologic recognition [5].

Given its upstream role in immune regulation, CD47 has emerged as a promising therapeutic target with the potential to bridge innate and adaptive immunity [6]. This is especially relevant for immunologically “cold” tumors that lack sufficient T-cell infiltration and respond poorly to programmed cell death protein 1 (PD-1)/programmed death-ligand 1 (PD-L1) blockade [7].

Despite this strong rationale, clinical translation has been more complex than anticipated. However, clinical translation has been hampered by on-target/off-tumor toxicities (notably anemia and thrombocytopenia), suboptimal pharmacokinetics, and modest single-agent efficacy, which together have tempered initial enthusiasm [8]. To address these barriers, several next-generation strategies are under active investigation. SIRPα-targeted agents seek to bypass direct CD47 engagement and may reduce hematologic toxicity [9]. Bispecific antibodies pair CD47 blockade with tumor-restricted antigens (e.g., CD20, EpCAM) to improve selectivity, while conditionally active therapeutics are engineered to activate preferentially within the tumor microenvironment, thereby widening the therapeutic index and minimizing systemic exposure [10]. Together, these innovations represent a shift toward more clinically translatable approaches that address the limitations of first-generation CD47 inhibitors [11].

Over the past decade, this rationale has driven the development of multiple anti-CD47 therapeutics, including CD47 monoclonal antibodies (e.g., magrolimab, letaplimab, lemzoparlimab, ligufalimab (AK117), maplirpacept, and AUR103), SIRPα-Fc function protein (e.g., evorpacept (ALX148), IMM01, BYON4228), and bispecific antibodies (e.g., IBI322, TG1801, IMM0306), among others [12].

However, clinical experience with CD47 inhibition has been more complex than anticipated [13–15]. In July 2025, Pfizer terminated a Phase II trial of its investigational CD47-targeting fusion protein maplirpacept (PF-07901801) in combination with tafasitamab and lenalidomide for relapsed or refractory diffuse large B-cell lymphoma (DLBCL), citing an inability to recruit the planned number of subjects after enrolling only six patients since August 2023. The decision was explicitly stated to be unrelated to safety or efficacy concerns (ClinicalTrials.gov Identifier: NCT05626322; last updated 29 June 2025). This follows a series of high-profile disappointments in the CD47 space, most notably Gilead’s complete discontinuation of magrolimab after multiple trial holds, including the termination of a Phase 3 study in higher-risk myelodysplastic syndrome (MDS) for futility (Gilead press release, 2023) and the termination of other ongoing MDS trials following cessation of magrolimab development (ClinicalTrials.gov Identifier: NCT05835011; last updated 02 October 2024).

Together with other setbacks, these events highlight not only the biological and clinical complexities of CD47 blockade, including anemia from red blood cell clearance but also strategic and operational challenges in trial design, patient selection, and combination approaches. Collectively, these developments underscore the urgent need to re-evaluate CD47-targeted strategies. This commentary aims to contextualize recent clinical outcomes, dissect mechanistic insights, refine their clinical translation, and outline future directions for advancing CD47 blockade into durable, safe, and effective cancer immunotherapies.

## 2 Mechanistic Insights: Beyond the “Don’t Eat Me” Signal

The CD47-SIRPα interaction constitutes a multifaceted checkpoint at the interface of innate immunity [16]. CD47 is a transmembrane protein that binds the extracellular immunoglobulin variable (IgV) domain of SIRPα, an inhibitory receptor expressed predominantly on myeloid cells [17]. Upon ligand engagement, SIRPα’s intracellular immunoreceptor tyrosine-based inhibitory motifs (ITIMs) become phosphorylated, recruiting the SH2-domain containing phosphatases Src homology region 2 domain-containing phosphatase-1 (SHP-1) and Src homology region 2 domain-containing phosphatase-2 (SHP-2) [18]. These phosphatases dephosphorylate key effectors required for actin remodeling, effectively halting phagocytic-cup formation and target engulfment [19].

Importantly, this pathway not only suppresses macrophage phagocytosis but also limits antigen uptake and presentation by DCs [4,20]. Because antigen presentation is essential for initiating T-cell responses, CD47 functions as a pan-immune checkpoint, impairing both innate and adaptive immunity [2,6]. This provides strong rationale for therapeutic inhibition, particularly in tumors with poor T-cell infiltration or loss of major histocompatibility complex (MHC) expression.

## 3 Clinical Setbacks: Lessons from Anemia, Selectivity, and Limited Efficacy

Despite compelling biological rationale, CD47-targeted therapies have encountered significant clinical hurdles. A primary concern is on-target, off-tumor toxicity resulting from CD47 expression on red blood cells (RBCs) [21,22]. Antibodies with unmodified Fc domains can opsonize RBCs, causing anemia and dose-limiting toxicities that complicate safe and sustained treatment [23]. Beyond RBCs, additional on-target/off-tumor effects warrant consideration. These include thrombocytopenia due to platelet targeting, potential involvement of hematopoietic stem cells, and risks of cytokine release syndrome (CRS) or other immune-related adverse events in certain contexts, particularly in combination regimens [24,25].

In addition, monotherapy trials, particularly in solid tumors, have generally shown modest efficacy [26,27]. This limited activity likely reflects redundancy within the tumor microenvironment (TME), where alternative immune-evasion pathways compensate for CD47 blockade [1]. The absence of robust predictive biomarkers further obscures whether poor responses stem from intrinsic resistance or suboptimal patient selection [8].

**Figure 1:**
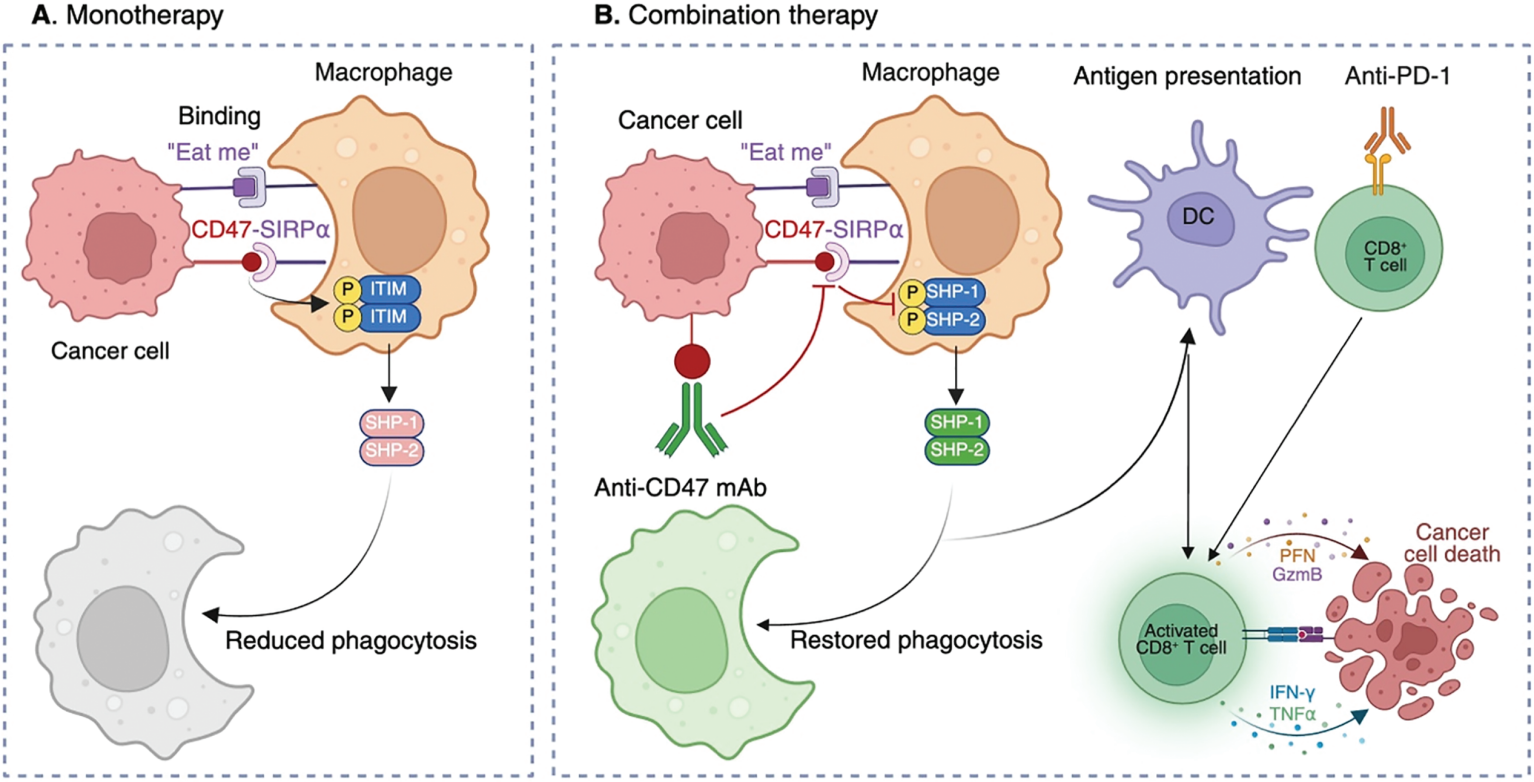
Mechanisms of CD47-mediated immune evasion and restoration of antitumor immunity through combination therapy. (**A**) Monotherapy: Tumor cells overexpress CD47, which binds SIRPα on macrophages. This interaction triggers phosphorylation of ITIMs in SIRPα, leading to recruitment of SHP-1 and SHP-2 and inhibition of cytoskeletal rearrangement, thereby suppressing phagocytosis despite “eat me” signals. (**B**) Combination therapy: Anti-CD47 monoclonal antibodies (mAbs) block the CD47-SIRPα interaction, preventing ITIM phosphorylation and SHP-1/SHP-2 recruitment. Macrophage phagocytosis is restored, enabling tumor cell engulfment and subsequent antigen presentation by DCs. Antigen-loaded DCs prime CD8^+^ cytotoxic T cells, which mediate tumor killing through perforin (PFN), granzyme B (GzmB), interferon-γ (IFN-γ), and tumor necrosis factor-α (TNF-α). Concomitant PD-1 blockade further enhances T-cell effector function. In parallel, myeloid reactivation supports recruitment of natural killer (NK) cells, contributing to direct tumor cell lysis and reshaping of the TME toward a pro-inflammatory, antitumor state. This figure originally conceived and created by the authors using BioRender (BioRender, Toronto, ON, Canada; https://app.biorender.com).

These challenges do not negate CD47 as a therapeutic target; rather, they highlight the need for more refined approaches that integrate tumor context, rational combinations, and biomarker-driven precision [28]. To visualize these concepts, Fig. 1 depicts the dual roles of the CD47-SIRPα axis in tumor immune evasion and the restoration of antitumor immunity through combination strategies.

To complement this discussion, Table 1 summarizes key CD47-targeted agents, their modalities, sponsors, indications, and trial outcomes, highlighting the patterns of both progress and discontinuation across the clinical landscape. This structured overview underscores the translational challenges of CD47 blockade and sets the stage for mechanistic considerations illustrated in Fig. 1.

**Table 1:** Key CD47-targeted agents in clinical development

Agent	Modality/ strategy	Company/ sponsor	Primary indication(s)	Clinical trials	Status
Maplirpacept (PF07901801)	Fusion protein (CD47-blocking decoy)	Pfizer	Relapsed/Refractory DLBCL, Multiple myeloma (MM)	NCT03530683	Terminated
Evorpacept (ALX-148)	SIRPα-Fc fusion protein (inert Fc)	ALX Oncology	AML (ASPEN-05)	NCT04755244	Terminated
			B-cell Non-Hodgkin Lymphoma	NCT05025800	Active, not recruiting
			Head and neck cancer	NCT04675294	Active, not recruiting
				NCT04675333	Active, not recruiting
				NCT05787639	recruiting
			Gastric/Gastroesophageal junction cancer	NCT05002127	Active, not recruiting
Magrolimab	Monoclonal antibody (anti-CD47)	Gilead Sciences	Acute Myeloid Leukemia (AML)	NCT04435691	Terminated
				NCT05829434	Withdrawn
				NCT05367401	Withdrawn
				NCT05079230	Terminated
				NCT05823480	Withdrawn
				NCT04778397	Terminated
				NCT03922477	Terminated
				NCT02678338	Completed
			Myelodysplastic Syndromes (MDS)	NCT04313881	Terminated
				NCT05835011	Terminated
				NCT05829434	Withdrawn
				NCT05367401	Withdrawn
				NCT05823480	Withdrawn
				NCT02678338	Completed

## 4 Strategic Innovations in CD47-Based Therapy

Several approaches are being developed to overcome the limitations of first-generation CD47 inhibitors. Combination immunotherapy is among the most promising: co-blockade of CD47 with immune checkpoints such as PD-1, v-domain immunoglobulin suppressor of T cell activation (VISTA), or CD24 can synergistically restore phagocytosis, enhance antigen presentation, and reinvigorate exhausted T cells [29–31]. Such dual-targeting strategies are particularly appealing in tumors employing multiple, redundant layers of immune suppression [32].

Bispecific antibodies represent another strategy. By pairing CD47 blockade with tumor-specific targets (e.g., CD20 or EpCAM), these agents achieve selective activity on malignant cells while sparing RBCs and other healthy tissues. Early data suggest improved safety and specificity with this approach [33,34]. Likewise, nanoparticle-based delivery systems can localize CD47 inhibitors to the TME, enhancing the therapeutic index by reducing systemic exposure [35,36].

Targeting the receptor side of the axis is also gaining traction. SIRPα-directed agents including decoy receptors and fusion proteins, bypass the “red-cell sink” and directly activate myeloid cells, offering a potentially broader therapeutic window [37–39]. Several candidates are now advancing through early-phase clinical trials [40,41].

Rational integration with chemotherapy provides additional opportunities. Cytotoxic agents can upregulate CD47 in response to stress, sensitizing tumors to blockade [42]. Sequence-dependent combinations may be particularly relevant in diseases like ovarian cancer, where chemotherapy remains the frontline therapy [41,43].

Other innovative antibody formats are also being engineered. Fc-silencing and Fc-tuning reduce off-tumor effector functions; extended half-life variants and intratumoral delivery strategies provide spatial control of immune activation and may mitigate hematologic toxicity [44,45]. Novel constructs such as diphtheria toxin-based bivalently immunotoxins, expressed through yeast-based systems, have also demonstrated high specificity and potent efficacy against CD47^+^ cancers with minimized off-target binding [46].

Finally, dual-targeting strategies are emerging as a particularly promising avenue. Combining CD47 inhibition with pro-phagocytic “eat-me” signals, such as calreticulin (CALR), can amplify macrophage-mediated clearance of tumor cells. Similarly, pairing CD47 blockade with DNA damage response therapies, including poly (ADP-ribose) polymerase (PARP) inhibitors, has shown synergistic potential by promoting immunogenic stress responses and facilitating tumor cell recognition [47,48]. These approaches may be especially valuable in immunologically “cold” tumors lacking endogenous “eat-me” signals, thereby providing a rationale for rational dual-targeting combinations in future clinical development.

## 5 Future Perspectives: Contextual Precision in CD47 Blockade

The next generation of CD47-targeted and other precision immunotherapies will hinge on optimized drug design, rigorous patient selection, and context-specific application. A central challenge is identifying which morphologically, genetically, and metabolically heterogeneous tumors are functionally dependent on CD47-mediated immune evasion [3,49]. Advanced technologies such as single-cell profiling, spatial-transcriptomics, and special proteomics can delineate immune phenotypes and uncover predictive biomarkers [50].

Spatially resolved technologies, including multiplex immunohistochemistry (mIHC) and co-detection by indexing (CODEX), add crucial context by assessing not only macrophage abundance but also their polarization state (M1 vs. M2), proximity to tumor cells, and co-localization with T cells [51]. Tumors enriched with M2-like, SIRPα^+^ macrophages at the invasive margin are more likely to benefit from CD47 blockade than those with sparse or M1-polarized macrophages [52]. Integrating these spatial metrics into biomarker development will refine patient stratification and guide rational therapeutic combinations [53].

Companion diagnostics incorporating CD47 expression levels, SIRPα isoforms, and myeloid signatures may further refine patient selection [54,55]. Such approaches could follow a stepwise process: baseline clinical evaluation to confirm diagnosis and associated symptoms, genetic risk stratification with mutation testing and identification of driver mutations, as well as immune transcriptomic profiling with comprehensive gene activity analysis, culminating in clustering by immune activity state (hyperactive, moderate, no active) [56,57].

The TME must also guide therapeutic choices. Macrophage-rich but T-cell-excluded tumors may benefit more from CD47 modulation than highly inflamed lesions dominated by lymphocytes [58]. Understanding each tumor’s immunologic “terrain” will be key to selecting effective combinations, whether with PD-1/PD-L1 antibodies, CD40 agonists, stimulator of interferon gene (STING) agonists, radiotherapy, or molecularly targeted drugs [59,60].

Clinical trial design should evolve accordingly. Adaptive frameworks incorporating real-time immune monitoring, pharmacodynamic biomarkers, and built-in combination arms can generate clearer efficacy signals and accelerate optimization [61,62]. Endpoints should extend beyond response rates to capture immune reprogramming within the TME [63,64]. In chronic lymphocytic leukemia (CLL), MDS, and multiple myeloma (MM), patient-oriented endpoints, including quality of life and patient-reported outcomes, will be essential to define the long-term value of CD47 blockade [65].

## 6 Conclusion

The CD47-SIRPα axis remains a compelling and versatile target in cancer immunotherapy. Recent clinical disappointments reflect the complexity of immune modulation rather than invalidation of the target itself. Moving forward, success will depend on strategic refinements of antibody engineering to minimize hematologic toxicity, biomarker-driven patient selection, and rational combinatorial regimens that exploit tumor context. The promise of CD47-targeted therapy lies not in questioning its relevance, but in applying it with precision: the right combinations, in the right patients, at the right time.

## Data Availability

Not applicable.

## Abbreviations

AML: Acute myeloid leukemia
CALR: Calreticulin
CD47: Cluster of differentiation 47
CLL: Chronic lymphocytic leukemia
CODEX: Co-detection by indexing
CRS: Cytokine release syndrome
DC: Dendritic cell
DLBCL: Diffuse large B-cell lymphoma
EpCAM: Epithelial cell adhesion molecule
GzmB: Granzyme B
IFN-γ: Interferon-gamma
ITIM: Immunoreceptor tyrosine-based inhibitory motif
mAb: Monoclonal antibody
MDS: Myelodysplastic syndrome
mIHC: Multiplex immunohistochemistry
MHC: Major histocompatibility complex
MM: Multiple myeloma
NK: Natural killer
PARP: Poly (ADP-ribose) polymerase
PD-1: Programmed cell death protein 1
PD-L1: Programmed death-ligand 1
PFN: perforin
RBC: Red blood cell
SHP-1: Src Homology region 2 domain-containing Phosphatase-1
SHP-2: Src Homology region 2 domain-containing Phosphatase-2
SIRPα: Signal regulatory protein alpha
STING: Stimulator of interferon genes
TME: Tumor microenvironment
TNF-α: Tumor necrosis factor-alpha
VISTA: v-domain immunoglobulin suppressor of T cell activation
